# The Effect of Chemical Disinfectants on Maxillofacial Silicone With the Addition of Silver Nanoparticles: An Original Research

**DOI:** 10.7759/cureus.66484

**Published:** 2024-08-08

**Authors:** Vedant Pathak, Seema R Kambala, Tanvi Jaiswal, Anjali Bhoyar, Mithilesh Dhamande

**Affiliations:** 1 Prosthodontics, Sharad Pawar Dental College and Hospital, Datta Meghe Institute of Higher Education and Research, Wardha, IND

**Keywords:** surface roughness, shore a hardness, color stability, silver nanoparticles, maxillofacial silicone

## Abstract

Background and objective

Silicone has emerged as the most widely accepted material for facial prosthesis fabrication. However, silicone materials have certain limitations. Several techniques have been investigated to lessen the degradation of the polymer, such as the use of nanoparticles and nano-oxides, etc. In this study, we aimed to evaluate the effect of various chemical disinfectants on color stability, hardness, and surface roughness of maxillofacial silicone, after the addition of silver nanoparticles.

Materials and methods

This was an in vitro study carried out in the Department of Prosthodontics, Sharad Pawar Dental College and Hospital; 80 samples of maxillofacial silicone incorporated with silver nanoparticles (in a concentration of 20 ppm) were fabricated in a mold of 3 x 10 mm dimension disc. The samples were then tested for surface roughness (using a digital roughness tester), Shore A hardness (using a durometer), and color stability (using a spectrophotometer). The samples were then classified into four groups according to various disinfectants used: sodium hypochlorite (1% w/w), chlorhexidine gluconate (0.2%), and neutral soap, and distal water was deemed the control group. After 48 hours, the samples underwent retesting to assess for changes in readings under the same parameters (i.e., surface roughness, Shore A hardness, and color stability) to obtain results, i.e., the samples were tested after fabrication, before immersion, and 48 hours after immersion in disinfectants.

Results

When taking into account the surface roughness, the maximum roughness value was observed in the sodium hypochlorite group and the least roughness value in distilled water (mean % change of 38.359 to negligible change in the distilled water group). As for the Shore A hardness, the maximum hardness value was seen in the sodium hypochlorite group and the least hardness value in distilled water (mean % change of 15.780 to 2.125 in distilled water). Regarding color stability, the maximum increase in color values was seen in the sodium hypochlorite group (mean: 2.4) followed by the neutral soap group (mean: 1.653); the chlorhexidine gluconate group (mean: -0.287) showed the maximum decrease in color value from the initial to the final phase.

Conclusions

Based on our findings, surface roughness altered the most when samples were immersed in 1% sodium hypochlorite disinfectant and the least when samples were immersed in neutral soap disinfectant. Shore A hardness altered the most when samples were immersed in 1% sodium hypochlorite disinfectant, but altered the least when samples were immersed in neutral soap disinfectant. Color stability altered the most when samples were immersed in neutral soap disinfectant, but altered the least when samples were immersed in 0.2% chlorhexidine gluconate. Disinfection with neutral soap seems to lead to fewer changes in physical properties (i.e., surface roughness and Shore A hardness) and hence is recommended as a disinfectant for silicone prosthesis. However, our study also showed that 0.2% chlorhexidine gluconate had the least effect on the parameter of color stability, and hence it could be the disinfectant of choice for prostheses with high esthetic requirements.

## Introduction

Since its introduction in 1960, silicone has become the most popular and clinically approved material for the creation of facial prostheses thanks to its biocompatibility, physical and mechanical qualities, and ease of manipulation. Patients feel comfortable and well-cared for because of its texture, which is similar to human skin [1]. Nevertheless, silicone materials have certain drawbacks, such as a shorter lifespan brought on by color instability and early deterioration, which alter the texture and cause edges to fit poorly and lose tear strength. As nanotechnology has advanced, elastomers have been strengthened by the addition of nanoparticles. Because of their higher surface-area-to-volume ratio, nanoparticles differ from their macroparticle counterparts in terms of physical, chemical, and biological properties [2].

Silver nanoparticles incorporated in silicone maxillofacial material have been used to serve as antifungal agents and demonstrate biocompatibility, yet their impact on material physical, mechanical properties, and color stability remains inadequately tested [3,4]. Therefore, this present study was carried out to evaluate the effect of chemical disinfectants on color stability, hardness, and surface roughness of maxillofacial silicone, after the addition of silver nanoparticles.

## Materials and methods

The research was conducted in the Department of Prosthodontics, Sharad Pawar Dental College and Hospital. Materials used included maxillofacial silicone (M 511 room temperature vulcanizing silicone, Factor II Inc, Technovent limited, 5 York Park, Bridgend UK) and silver nanoparticles (2%w/w, Ultrananotech, Bangalore, India). Chemical disinfectants included 1% sodium hypochlorite, 0.2% chlorhexidine gluconate, and neutral soap. All samples were fabricated as per specification by using a customized mold.

Materials

The following tools were also used: a double-beam UV-visible spectrometer for measuring spectral reflectance in the visible range of wavelengths 200-800 nm; an ultraviolet reflectance spectrometer (Labman Scientific Instruments, Chennai, India) for color stability; a durometer (MGW Precision Shore A Hardness Tester Durometer, MGW Precision, Mumbai, India) for Shore A hardness; and a digital roughness tester (portable surface roughness tester, Surftest SJ-410 Series, Mitutoyo, Kawasaki, Japan) for surface roughness.

Methodology

Fabrication of Samples

A customized metal mold was fabricated, which comprises 10 slots each of dimension 3 x 10 mm. Dispensation of a 10:1 W/w ratio of base and catalyst of RTV maxillofacial silicone (i.e., 10 grams of part A and 1 gram of part B) was done. To create the test specimens, silicone elastomer and silver nanoparticles were combined with elastomer primer (part B). Nano powder, with a concentration of 20 ppm, was added, mixed in a glass beaker, and placed in an ultrasonic vibrator to ensure that the nanoparticles were distributed properly. Per the manufacturer's instructions, part B of the elastomer material was then combined with part A. Careful spatulation for 10 minutes was done as per the manufacturer's recommendation to obtain a uniform mix free of air bubbles. Disc-shaped samples were fabricated, which comprised silver nanoparticles (20ppm), and maxillofacial silicone (10:1) was formulated.

The formulated material was then packed into the custom-made metal mold and the mold was covered with a lid and tightened with screws and kept for 24 hours; 100 samples were fabricated and 80 error-free samples were selected. They were classified into four groups according to the disinfectant used for the study: 20 samples each in sodium hypochlorite, chlorhexidine gluconate, neutral soap, and distilled water). The samples were then tested for all the parameters: surface roughness, Shore A hardness, and color stability. The categorized samples were immersed in the disinfectants with 20 samples in each group. After 48 hours of immersion, the samples underwent retesting to assess for changes in readings under the same parameters to obtain results.

Testing of the samples

The following tests were conducted on the samples: surface roughness, Shore A Hardness, and color stability.

A portable digital roughness tester (Surftest SJ-410 Series, Mitutoyo) with a measurement course of 6 mm and an accuracy of 0.01 µm was utilized for the surface roughness test. As opposed to the Shore, rubber materials are subjected to a hardness test that is carried out in compliance with ASTM D2240 standards. For soft vulcanized rubber and thermoplastic elastomers, a durometer is usually used. On the other hand, a spectrophotometer was used to collect data on color changes (∆E), which were ascertained from the samples' initial and final reflectance values by the spectrometer. A UV filter was set to 100% UV and a tungsten lamp with D50 standard illumination was used with a 2° viewing angle.

Following that, for a full 48 hours, every sample from the four groups (sodium hypochlorite, chlorhexidine gluconate, neural soap, and distilled water) was submerged in the aforementioned disinfecting solutions. Samples were retested for each of the three parameters - surface roughness, Shore A hardness, and color stability -after disinfection. To simulate a year of clinical use, the silicone specimens were disinfected for 48 hours using various types of disinfecting agents; 48 hours over 360 days is equal to eight minutes of treatment per day. Thus, the samples were subjected to a 48-hour immersion in a disinfecting solution as part of the chemical disinfection process.

The findings were documented and the comparison was done between initial and final values of surface roughness, Shore A hardness, and color stability. Intergroup comparison was also done between various disinfectants (1% sodium hypochlorite, 0.2% chlorhexidine, neutral soap, and distilled water).

## Results

**Figure 1:**
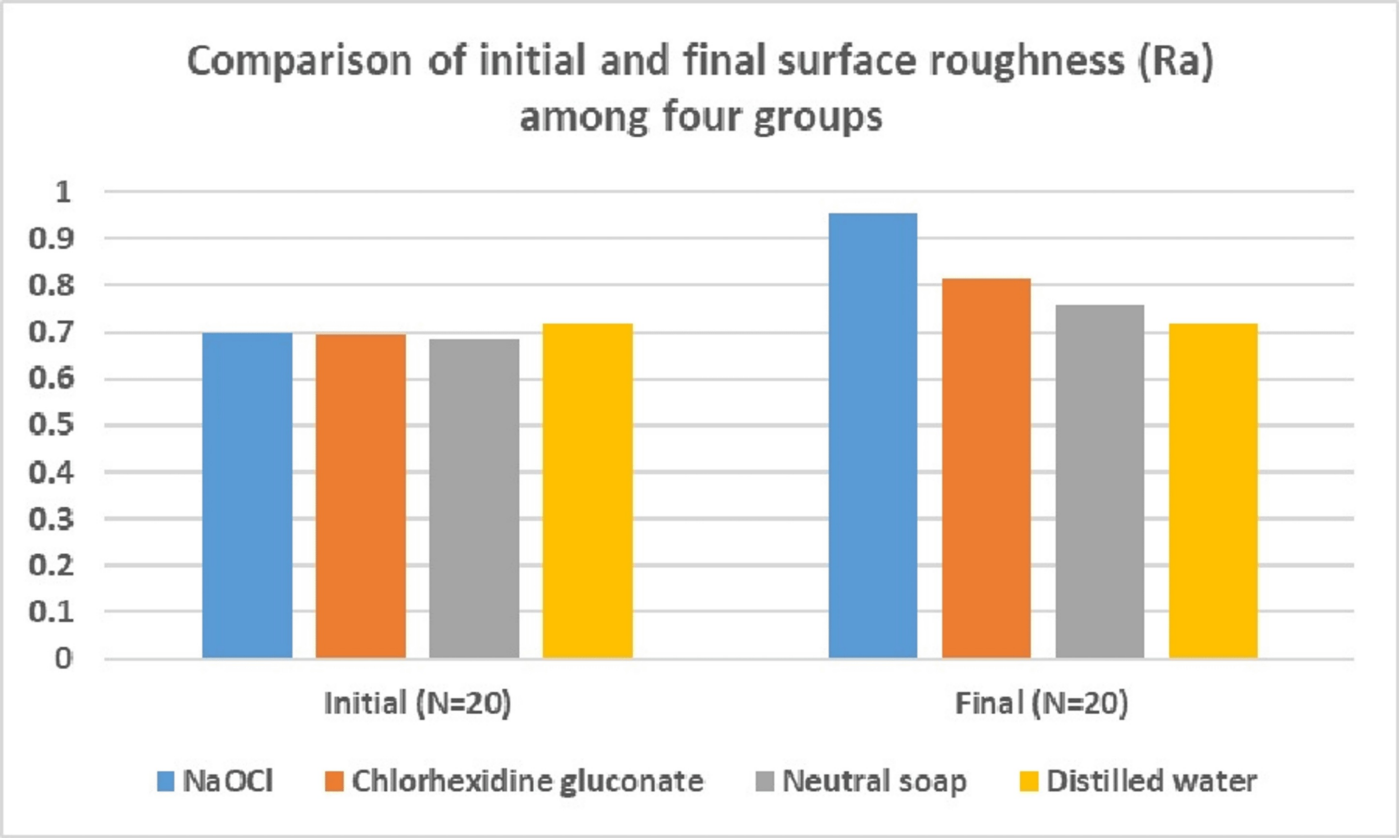
Comparison of the initial and final surface roughness (Ra) among the four groups NaOCl: sodium hypochlorite

A comparison of initial surface roughness showed a non-significant difference among the four groups (Table 1). The final surface roughness evaluation showed a maximum roughness value in the sodium hypochlorite group and the lowest roughness value in distilled water. The difference in the final surface roughness of the four groups was significant (Figure 1).

**Table 1:** Comparison of the initial and final surface roughness (Ra) among the four groups *Statistically significant NS: not significant

Group	Initial (N=20)		Final (N=20)	
	Mean	Std. Deviation	Mean	Std. Deviation
Sodium hypochlorite	0.698	0.133	0.952	0.132
Chlorhexidine gluconate	0.696	0.159	0.813	0.163
Neutral soap	0.687	0.126	0.758	0.134
Distilled water	0.719	0.157	0.719	0.157
P-value	0.909 (NS)		<0.001*	

The results of the magnitude of change in the surface roughness value (in %) from the initial to final phase in each group are presented in Table 2. Except for distilled water, surface roughness value showed an increase in all the other three groups with sodium hypochlorite showing the highest increase (38.36%). The difference in the magnitude of change in the surface roughness value (in %) from the initial to final phase among the four groups was significant.

**Table 2:** Comparison of the change in surface roughness (in %) among the four groups *Statistically significant

Group	Mean % change	Std. Deviation	P-value
Sodium hypochlorite	38.359	15.104	<0.001*
Chlorhexidine gluconate	17.650	7.154	
Neutral soap	10.738	5.558	
Distilled water	0.000	0.000	

**Figure 2:**
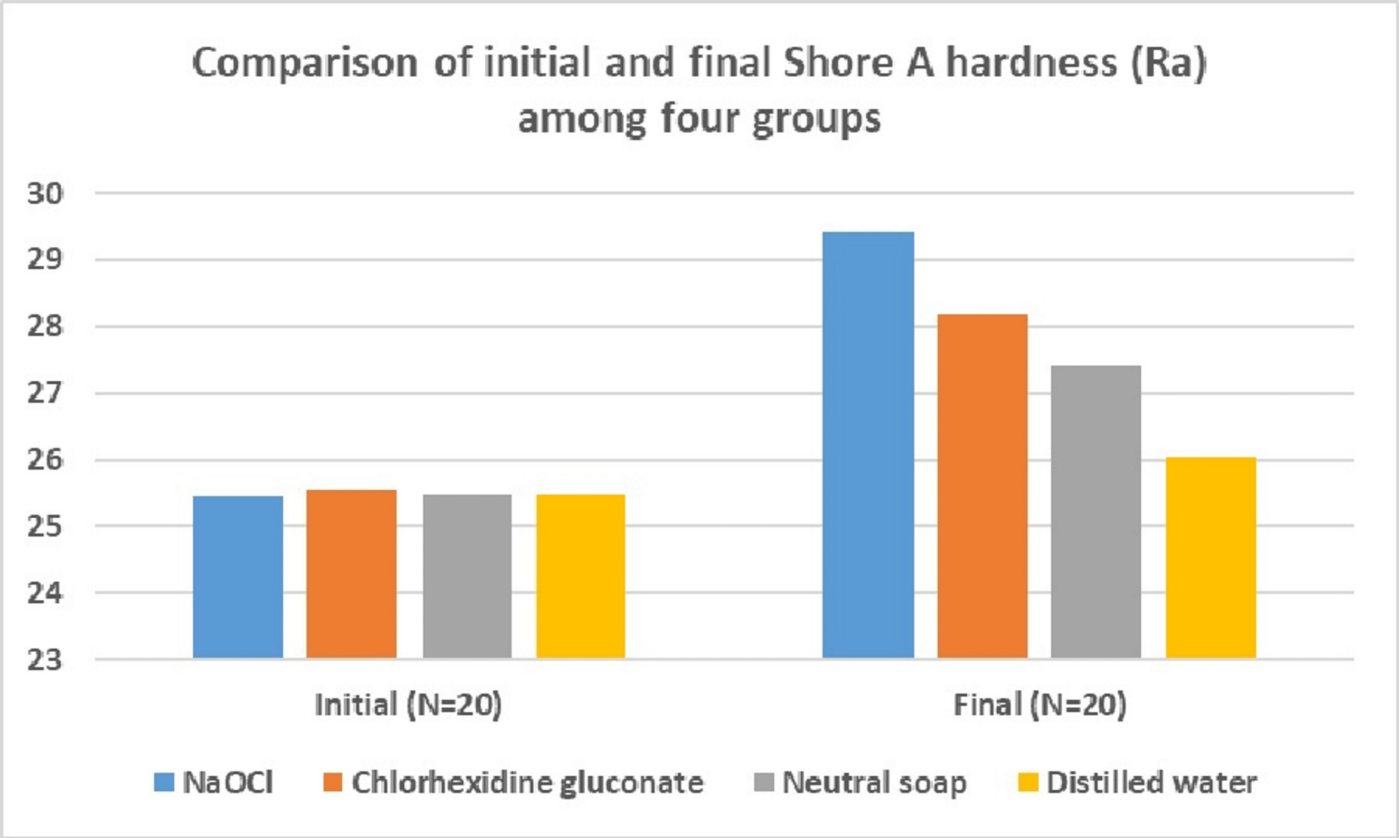
Comparison of the initial and final Shore A hardness among the four groups NaOCl: sodium hypochlorite

The comparison of initial Shore A hardness showed a non-significant difference among the four groups(Table 3). Final Shore A hardness evaluation showed a maximum hardness value in the sodium hypochlorite group and the least hardness value in distilled water. The difference in the final surface hardness among the four groups was significant (Figure 2).

**Table 3:** Comparison of the initial and final Shore A hardness among the four groups *Statistically significant NS: not significant

Group	Initial (N=20)		Final (N=20)	
	Mean	Std. Deviation	Mean	Std. Deviation
Sodium hypochlorite	25.450	3.000	29.400	3.102
Chlorhexidine gluconate	25.550	3.017	28.200	3.054
Neutral soap	25.500	3.517	27.400	3.378
Distilled water	25.500	3.887	26.050	4.045
P-value	1.000 (NS)		0.022*	

The results of the magnitude of change in the Shore A hardness value (in %) from the initial to the final phase in each group are presented in Table 4. The Shore A hardness value showed an increase in all the groups with sodium hypochlorite showing the highest increase (15.78%). The difference in the magnitude of change in the Shore A hardness value (in %) from the initial to final phase among the four groups was significant. 

**Table 4:** Comparison of the change in Shore A hardness among the four groups *Statistically significant

Group	Mean	Std. Deviation	P-value
Sodium hypochlorite	15.780	4.625	<0.001*
Chlorhexidine gluconate	10.566	3.933	
Neutral soap	7.659	3.566	
Distilled water	2.125	2.320	

**Figure 3:**
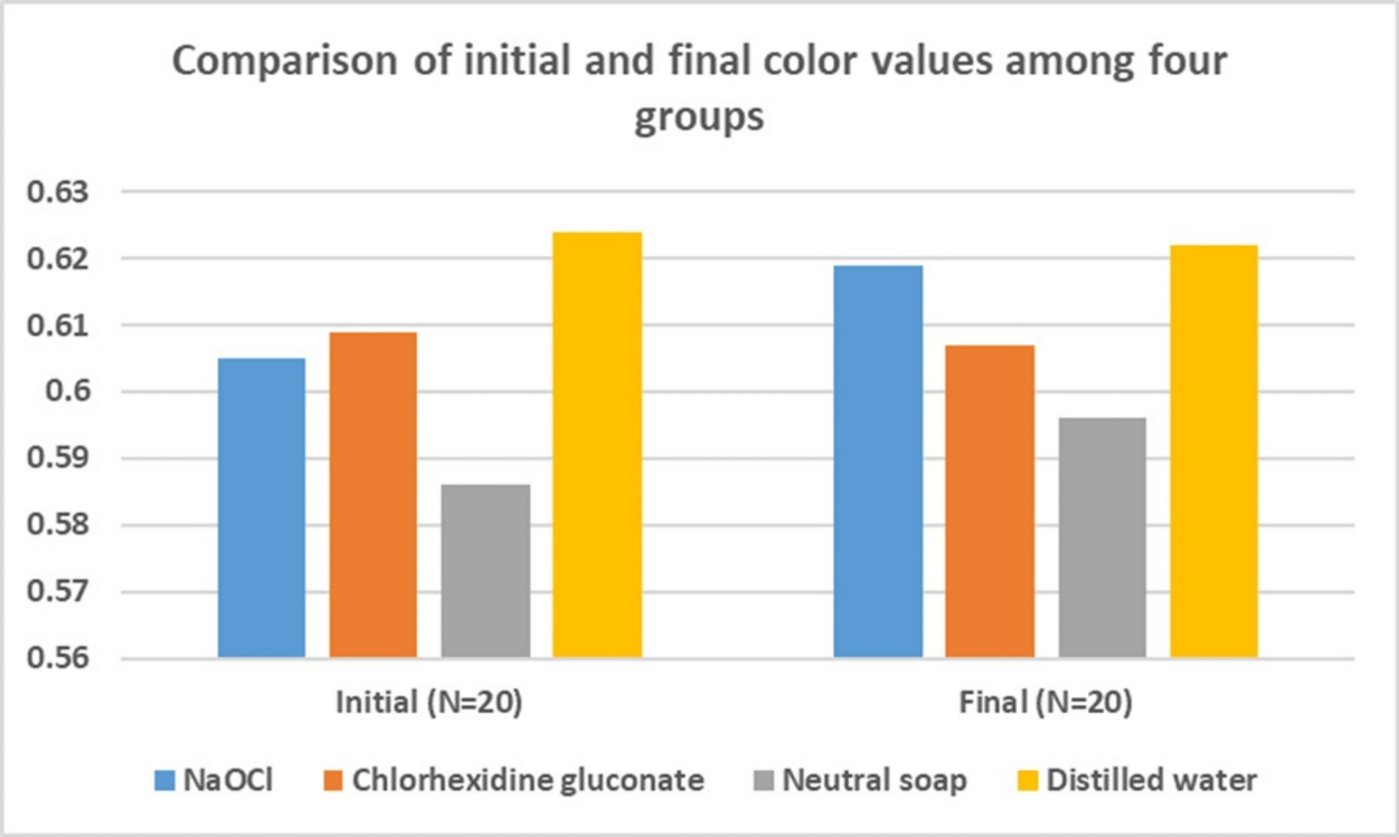
Comparison of the initial and final color values among the four groups NaOCl: sodium hypochlorite

A comparison of initial color values showed a non-significant difference among the four groups (Table 5). Similarly, a comparison of final color values showed a non-significant difference among the four groups (Figure 3).

**Table 5:** Comparison of the initial and final color values among the four groups NS: not significant

Group	Initial (N=20)		Final (N=20)	
	Mean	Std. Deviation	Mean	Std. Deviation
Sodium hypochlorite	0.605	0.084	0.619	0.081
Chlorhexidine gluconate	0.609	0.068	0.607	0.071
Neutral soap	0.586	0.060	0.596	0.053
Distilled water	0.624	0.085	0.622	0.080
P-value	0.591 (NS)		0.828 (NS)	

The magnitude of change in color values (in %) among the four groups is presented in Table 6. The maximum increase in color values was seen in the sodium hypochlorite group followed by the neutral soap group; the chlorhexidine gluconate group showed a maximum decrease in color value from the initial to the final phase. The difference in the magnitude of change in color values (in %) among the four groups was significant. 

**Table 6:** Comparison of the change in color values among the four groups *Statistically significant

Group	Mean	Std. Deviation	P-value
Sodium hypochlorite	2.414	4.727	0.034*
Chlorhexidine gluconate	-0.287	1.511	
Neutral soap	1.653	3.414	
Distilled water	-0.197	2.772	

## Discussion

Maxillofacial prostheses offer an alternative treatment option when surgical correction of maxillofacial defects is not feasible. Maxillofacial prosthetic materials are increasingly being used to treat functional and cosmetic deficiencies in patients with significant facial defects [5-7]. Maxillofacial materials must resemble human tissues regarding their mechanical and physical characteristics. These materials should maintain these characteristics for the entirety of their lifespan [8-10]. Key properties of maxillofacial materials include high color stability, minimal surface roughness change, and Shore A hardness.

Because silicone elastomers are clinically inert, strong, durable, easily manipulable, and have an adequate bond to underlying materials, they are currently the material of choice for maxillofacial prostheses. Silicone offers several benefits, including enhanced patient comfort, durability, ease of coloring and manipulation, strength, and a texture that almost exactly resembles human tissue [11,12]. However, silicone's quick deterioration of physical properties and unstable color are the reasons for its short shelf life in maxillofacial prostheses. Its difficult repair procedure also prevents it from being extensively used in this industry [13]. For example, it might have different textures and uneven edges due to the decreased tear strength.

The modifications are directly influenced by the patient's hygiene, handling practices, cleaning supplies, and the kinds of exposures the prosthesis receives, including temperature changes, UV and solar radiation, moisture, air pollution, and climate changes. The interactions between silica fillers, polymer chains, and the environment may be impacted during service, necessitating prosthesis replacement [14-16]. Various strategies are employed to halt this degradation of the polymer [17,18]. One of the main areas of advancing science, nanotechnology, has witnessed a recent trend involving the use of nanoparticles. Because of their high surface-to-volume ratio, nanoparticles have different physical, chemical, and biological properties from their macroparticle counterparts.

This study looks at how different chemical disinfectants affect the color stability, Shore A hardness, and surface roughness of maxillofacial silicone that contains silver nanoparticles. All samples were treated with silver nanoparticles and then subjected to various disinfectants: distilled water (as the control group), sodium hypochlorite, chlorhexidine gluconate, and neutral soap. To simulate a year of clinical use, the silicone specimens were disinfected for 48 hours using various types of disinfecting agents; 48 hours over 360 days is equal to eight minutes of treatment per day. Thus, the samples were subjected to a 48-hour immersion in a disinfecting solution as part of the chemical disinfection process [19-22].

The sodium hypochlorite group had the highest surface roughness value (mean change: 38.359%) in the current study, while the neutral soap group had the lowest roughness value. Except for distilled water, all groups had a rise in the percentage of surface roughness change from the initial to the final phase; sodium hypochlorite showed the largest increase (38.36%). This study supports the 2009 findings of Goiato et al., which indicate that increased roughness is not related to chemical disinfectants but rather results from the material's high filler content [23]. As a result, nanoparticles increase the filler content as well. On the other hand, our results contradict those of Babu et al. [24], who reported that sample surface roughness decreased following the disinfection period. The following samples were used in their research: neutral soap, accelerated aging, 4% chlorhexidine, and FittyDent tablets.

In this study, a pairwise comparison of initial and final Shore A hardness among the four groups showed a significant increase in the final Shore A hardness value in the sodium hypochlorite group (mean change: 15.78%), followed by the chlorhexidine gluconate group (10.566%), and neutral soap group (7.659%). Our findings align with the findings of Lanzara et al. (2019) [25], Guiotti et al. [26], Guiotti et al. [27], Babu et al. [24], and others, which propose that enhanced cross-linking of polymeric chains and ongoing polymerization processes cause hardness to rise. Guiotti et al. attributed the increased hardness to the evaporation of volatile by-products [27]. Compared to the group that did not receive disinfection, our study shows significant differences, which contrast with the findings of Goiato et al. (2009) [23]. We discovered that the physical characteristics of the material, such as increased absorption and solubility, could be harmed by chemical cleaning agents, particularly when samples are submerged in a sodium hypochlorite solution.

The prosthesis's lifespan is limited by silicone elastomer color degradation, which frequently requires the creation of a new prosthesis within six months to a year [28,29]. Certain environmental factors like temperature, exposure to water, and sun radiation can cause early color deterioration. Initial tests in the current study revealed no significant differences between any of the four groups, and the final results also revealed no significant differences in color value between any of the groups. However, the sodium hypochlorite group showed the greatest color instability (mean change: 7.659%), with the neutral soap group showing the least amount of instability (1.653%). In contrast, the group exposed to chlorhexidine gluconate showed very little color instability (-0.287%) from the first to the last phase. The results were similar to those of distilled water, which acted as a control group.

The surface properties of polymers can be linked to changes in color and hardness, which could result in the release of particular compounds into water or disinfectant solutions from the polymer matrix. Chemical disinfectants react with silicone, causing elastomer breakdown and chain bond disruption that compromises color stability. The results of our investigation support the findings of Hatamleh et al.'s study [30], which also highlights related effects on color stability. The color shift that was noticed in the neutral soap after it was disinfected with hypochlorite might have resulted from the digital friction disinfection process, which was supposed to remove pigments (nanoparticles) from the material's outer layer.

Our study has a few limitations, primarily its observational, in-vitro design. Hence, we recommend in vivo studies to gain deeper insights into the topic. Further studies can be planned to evaluate other physical parameters like tensile strength, tear strength, etc.

## Conclusions

Clinicians can gain valuable insights from our findings regarding the physical characteristics of maxillofacial silicone and how disinfection protocol affects it. Despite the limitations of the study, it can be concluded that sodium hypochlorite should not be used as a disinfectant for silicone maxillofacial prostheses because it increases surface hardness and roughness, which is surpassed by the neutral soap solution and chlorhexidine mouthwash. For color instability of the prosthesis, sodium hypochlorite ranks first followed by neutral soap solution. Furthermore, it is critical to consider the possibility that prolonged digital friction from using neutral soap could cause the prosthesis's surface to lose color. Patients should be advised against using neutral soap with scrubbing to disinfect a prosthesis.

## References

[1] Zayed SM, Alshimy AM, Fahmy AE (2014). Effect of surface treated silicon dioxide nanoparticles on some mechanical properties of maxillofacial silicone elastomer. Int J Biomater.

[2] Abou El-Nour KMM, Eftaiha A, Al-Warthan A, Ammar RAA (2010). Synthesis and applications of silver nanoparticles. Arab J Chem.

[3] Gangadharappa P, Rathore BS, Yaqoob A, Agrawal P, Patri G, Tejaswi CK, Kommuri S (2022). Effect of silver nanoparticle on properties of maxillofacial silicone elastomer material: an original research. Int J Health Sci.

[4] Kiat-Amnuay S, Mekayarajjananonth T, Powers JM, Chambers MS, Lemon JC (2006). Interactions of pigments and opacifiers on color stability of MDX4-4210/type A maxillofacial elastomers subjected to artificial aging. J Prosthet Dent.

[5] Jani RM, Schaaf NG (1978). An evaluation of facial prostheses. J Prosthet Dent.

[6] Yu R, Koran A, Craig RG (1980). Physical properties of maxillofacial elastomers under conditions of accelerated aging. J Dent Res.

[7] Lewis DH, Castleberry DJ (1980). An assessment of recent advances in external maxillofacial materials. J Prosthet Dent.

[8] Pesqueira AA, Goiato MC, Dos Santos DM, Haddad MF, Moreno A (2012). Effect of disinfection and accelerated ageing on dimensional stability and detail reproduction of a facial silicone with nanoparticles. J Med Eng Technol.

[9] Montgomery PC, Kiat-Amnuay S (2010). Survey of currently used materials for fabrication of extraoral maxillofacial prostheses in North America, Europe, Asia, and Australia. J Prosthodont.

[10] Mitra A, Choudhary S, Garg H, Jagdish HG (2014). Maxillofacial prosthetic materials- an inclination towards silicones. J Clin Diagn Res.

[11] Roberts AC (1971). Silicones for facial prostheses. Dent Pract Dent Rec.

[12] Aziz T, Waters M, Jagger R (2003). Analysis of the properties of silicone rubber maxillofacial prosthetic materials. J Dent.

[13] Huber H, Studer SP (2002). Materials and techniques in maxillofacial prosthodontic rehabilitation. Oral Maxillofac Surg Clin North Am.

[14] Kanter JC (1970). The use of RTV silicones in maxillofacial prosthetics. J Prosthet Dent.

[15] Nobrega AS, Andreotti AM, Moreno A, Sinhoreti MA, Dos Santos DM, Goiato MC (2016). Influence of adding nanoparticles on the hardness, tear strength, and permanent deformation of facial silicone subjected to accelerated aging. J Prosthet Dent.

[16] Roberts AC (1967). Facial reconstruction by prosthetic means. Br J Oral Surg.

[17] Wolfaardt JF, Chandler HD, Smith BA (1985). Mechanical properties of a new facial prosthetic material. J Prosthet Dent.

[18] Bayda S, Adeel M, Tuccinardi T, Cordani M, Rizzolio F (2019). The history of nanoscience and nanotechnology: from chemical-physical applications to nanomedicine. Molecules.

[19] Priyadarsini S, Mukherjee S, Mishra M (2018). Nanoparticles used in dentistry: a review. J Oral Biol Craniofac Res.

[20] Sharan J, Singh S, Lale SV, Mishra M, Koul V, Kharbanda P (2017). Applications of nanomaterials in dental science: a review. J Nanosci Nanotechnol.

[21] Andres CJ, Haug SP, Munoz CA, Bernal G (1992). Effects of environmental factors on maxillofacial elastomers: part I--literature review. J Prosthet Dent.

[22] Cevik P, Yildirim-Bicer AZ (2017). Effect of different types of disinfection solution and aging on the hardness and colour stability of maxillofacial silicone elastomers. Int J Artif Organs.

[23] Goiato MC, Pesqueira AA, Santos DM, Dekon SF (2009). Evaluation of hardness and surface roughness of two maxillofacial silicones following disinfection. Braz Oral Res.

[24] Babu AS, Manju V, Gopal VK (2018). Effect of chemical disinfectants and accelerated aging on maxillofacial silicone elastomers: an in vitro study. Indian J Dent Res.

[25] Lanzara R, Mandamparambil V, Kumar D (2022). Effect of disinfection on the physical properties of maxillofacial silicone material: a systematic review. Paripex Indian J Res.

[26] Guiotti AM, Goiato MC, Dos Santos DM (2016). Comparison of conventional and plant-extract disinfectant solutions on the hardness and color stability of a maxillofacial elastomer after artificial aging. J Prosthet Dent.

[27] Guiotti AM, Goiato MC, dos Santos DM (2010). Evaluation of the Shore A hardness of silicone for facial prosthesis as to the effect of storage period and chemical disinfection. J Craniofac Surg.

[28] Sonnahalli NK, Chowdhary R (2020). Effect of adding silver nanoparticle on physical and mechanical properties of maxillofacial silicone elastomer material-an in-vitro study. J Prosthodont Res.

[29] Morton RP, Izzard ME (2003). Quality-of-life outcomes in head and neck cancer patients. World J Surg.

[30] Hatamleh MM, Watts DC (2010). Mechanical properties and bonding of maxillofacial silicone elastomers. Dent Mater.

